# Alginate–Arabinoxylan Composite Films with Enhanced Mechanical Strength and Functional Properties for Potential Food Packaging Use

**DOI:** 10.3390/foods15061035

**Published:** 2026-03-16

**Authors:** Gargi Dandegaonkar, Ali Nawaz, Parikshit Goswami, Chenyu Du

**Affiliations:** 1 School of Applied Sciences, University of Huddersfield, Huddersfield HD1 3DH, UK; gargi.dandegaonkar@hud.ac.uk (G.D.); a.nawaz@hud.ac.uk (A.N.); 2 Technical Textiles Research Centre, University of Huddersfield, Queensgate, Huddersfield HD1 3DH, UK; p.goswami@hud.ac.uk

**Keywords:** biofilms, antimicrobial, shelf life, food packaging, edible film

## Abstract

The concern about plastic pollution drives the exploration of sustainable and environmentally friendly packaging materials. Alginate is a renewable, edible feedstock extracted from seaweed, which has been used for preparing edible biofilms. The major limiting factor in alginate biofilms wider application is that it is relatively weak in strength. This study explored a novel alginate composite biofilm prepared using alginate and maize bran derived arabinoxylans. In comparison with alginate alone, adding 2.5% *w*/*w* maize arabinoxylans increased the tensile strength of the film by 3.1 times. Using an optimized composition (2.5% alginate, 1% glycerol and 1.5% maize arabinoxylans), the tensile strength and elongation of the biofilm increased to 4.9 and 3.0 times that of alginate only biofilm to 6.88 ± 0.06 MPa and to 96.4 ± 9.9%, respectively. Interestingly, the water-holding capacity of biofilm increased from 5.5 times weight of water for 5 min for alginate alone biofilm to 27.6 times the weight of water for 50 min. When 0.5% clove essential oil was incorporated into the composite film, the biofilm exhibited excellent anti-microbial property, keeping raw meat free of bacteria for five days in both refrigerated and open environments. These results indicate that the alginate-based bio-composite film is a promising candidate for food packaging.

## 1. Introduction

The food industry is one of the largest users of plastic packaging. Around 36% of plastic produced worldwide is used for food and beverage packaging [1]. In the United Kingdom, it is estimated that approximately 2.2 million tons of plastic packaging are used annually. Traditionally, plastic has been widely used in the packaging industry due to its versatility, excellent mechanical properties—including high strength, durability, flexibility, barrier properties and cost-effective manufacturing [2]. However, the escalating plastic pollution crisis poses a significant challenge to modern society to transition away from non-renewable, non-biodegradable material. These concerns have driven a growing demand for sustainable and biocompatible alternatives to food packaging.

Recently there has been growing global interest in using edible biopolymers derived from renewable biomass to produce composite films. One prominent example of such biopolymer is sodium alginate (SA), which is sourced from brown algae and has an estimated annual production of 16.2 million tons annually [3]. But biofilms prepared using sodium alginate alone are weak and brittle, limiting its wider application. Adding gel forming monomers, such as polyacrylamide (PAM), poly (acrylic acid) (PAA), or a similar acrylate/methacrylate copolymer would improve the mechanical strength, but the resulting film is not edible, preventing its application for food packaging. To address this challenge, a few researchers investigated the feasibility of adding food-derived materials into alginate film to form stronger composite film, such as polysaccharides [4,5], proteins [6,7] and lipids [8]. In a recent report by Bhatia et al. [9], an edible packaging film formulated with 1.5% (*w*/*v*) SA, 1.5% (*w*/*v*) pectin, and cassia essential oil exhibited a tensile strength of 5.84 ± 0.23 MPa and an elongation of 122.87 ± 3.89%. However, for practical edible food-packaging applications—particularly for flexible wraps and films—mechanical requirements are generally higher, with tensile strengths typically expected in the range of 8–15 MPa and elongation values often above 150%, depending on the intended function (e.g., coating, wrapping, pouching). In another study, tensile strength of 1.38 ± 0.13 MPa was achieved for biofilm composed of 0.2% Boswellia sacra oleo gum resin, 1% cellulose, 1% alginate, 1% gelatin, which could not be considered good for food packaging application [10]. Furthermore, gelatin is an animal-based product and, hence, is not suitable for people following a vegetarian or vegan diet. Therefore, exploring low cost, plant based and abundant edible materials for forcing strong alginate films for food packaging applications is still a challenge.

Arabinoxylans (AX) are a group of edible polysaccharides that can be extracted from a wide range of lignocellulosic biomass, such as wheat bran, maize bran and sugarcane bagasse [11,12]. They are increasingly recognized for their potential in food application due to their bioactive properties, low cost, and applications as food functional ingredients, viscosity enhancers and gel forming agents [13]. Recently, Weng et al. [14] demonstrated the film forming ability of AX by mixing Maize AX with glycerol. Similarly, Alahmed and Simsek [15] synthesized AX-based films with the addition of sorbitol. These studies indicated AX could be a suitable additive to SA to form a strong SA-AX composite film.

In this study, novel bio-composite films composed of SA and AX were developed. Two different sources of AX—maize arabinoxylans (MAX) and wheat arabinoxylans (WAX)—were compared for tensile strength and elongation properties of the resulting films. The effects of SA concentration and MAX concentration, which exhibited higher tensile strength than WAX, were further examined in terms of tensile strength, elongation, thickness, moisture content, water-holding capacity, saline-holding capacity, and solubility of the resulting films. Finally, the addition of clove-essential-oil-based nano-emulsions into optimal films was analyzed for exhibition of antimicrobial property.

## 2. Materials and Methods

### 2.1. Materials

Food-grade SA (E401) was purchased from Special Ingredients via Amazon UK (CAS: 9005-38-3, London, UK). Commercial food grade WAX and MAX were kindly provided by Prof. Grant Campbell at the University of Huddersfield, characterized by Solomou et al. [16]. Glycerol, 99+%, pure, synthetic, from Fischer Scientific (CAS: 56-81-5|C_3_H_8_O_3_|92.09 g/mol) was used. The food grade Clove Essential Oil (C8392) was purchased from Sigma-Aldrich (d: 1.04 g/mL at 25 °C, CAS-No.: 8000-34-8, Waltham, MA, USA).

### 2.2. Bio-Composite Films

Bio-composite films were prepared by solvent casting according to Table 1 (all components reported as wt.% relative to SA). For each batch, SA was dissolved to 2.5% (*w*/*v*) in deionized water (2.5 g/100 mL) under magnetic stirring at 300 rpm for 2 h at 23 ± 2 °C (VELP Scientifica, Usmate Velate, Italy). When required, MAX/WAX (20–100 wt.%) was sprinkled into the vortex and mixed for 60 min, followed by dropwise addition of glycerol (50.4 wt.%) with an additional 30 min of stirring. The clove-oil nano-emulsion (CONE; 20 wt.%) was prepared immediately before use by sonicating clove oil:water:vegetable oil (1:8:1, *v*/*v*/*v*) with a probe sonicator (Vibra-Cell™, 130 W, 20 kHz) at 60% amplitude for 30 min using a 15 s on/10 s off duty cycle in an ice–water bath (<30 °C), then added last under gentle stirring for 15 min [17,18]. Exactly 25.0 g of the homogenized solution was weighed (KERN PFB 120-3) into 9 cm-diameter polystyrene Petri dishes (Fisher Scientific, Cat. No. FB0875712, Saint Louis, MO, USA), yielding a casting area of 63.6 cm^2^ and constant mass-per-area (0.393 g cm^−2^) for thickness control. Films were dried in a forced-air oven at 40 °C for 15 h, peeled, and conditioned 24 h at room temperature over silica gel before testing (Figure S1). Thickness was measured at five locations (center and four quadrants, ≥10 mm from edges) with a digital micrometer (1 µm resolution) and reported as mean ± SD (*n* = 5); batches deviating by >10% from target thickness were discarded and re-cast.

### 2.3. Characterization of the Films

- Mechanical Properties

The tensile strength and elongation at break of the film were analyzed using a TB400C Single Fibre Strength Tester (Testex, Zürich, Switzerland) fitted with a 10 N load cell. The films were conditioned for 24 h at 20 ± 1 °C, 65 ± 4% relative humidity. The film was cut in a 3.0 cm × 0.5 cm rectangle shape, which was mounted in the tensile grips. Testing was conducted at a constant speed of 10 mm min^−1^.

- Thickness

The thickness of the film was tested using a TF121C Digital Thickness Gauge (ATI Corporation Ltd., New Holland, PA, USA). Measurements were conducted at five distinct locations on each film. At least three films per type were tested.

- Moisture content

For the moisture content analysis, film samples were cut into 2.0 cm × 2.0 cm squares. Each sample was weighed to determine its initial mass and then dried in an oven at 100 °C for 24 h. After drying, the samples were weighed again to obtain their dry mass. The moisture content of the samples was calculated using the following equation:

$$Moisture\;content\;\left(\%\right)=\frac{\left(Initial\;weight\;of\;the\;sample-weight\;after\;drying\right)}{Initial\;weight\;of\;the\;sample}\;\times 100$$

- **Water-holding capacity and saline-holding capacity**

The water-holding capacity was evaluated using deionized water, while the saline-holding capacity was assessed using a saline solution prepared by dissolving 0.368 g CaCl_2_·2H_2_O and 2.298 g NaCl in 1 L of deionized water. Film samples (3.0 cm × 3.0 cm) were dried at 40 °C until constant weight and then placed in individual Petri dishes and incubated at 37 °C. For each test, an amount of swelling medium equivalent to 100 times the dry weight of the film was added to ensure complete immersion. At predetermined time intervals, samples were removed using tweezers, excess surface liquid was drained, and the film was lightly blotted with filter paper. The holding capacity (%) was calculated as follows:

$$Holding\;Capcity\left(\%\right)=\frac{\left(Wet\;weight\;of\;the\;sample-dry\;weight\;of\;the\;sample\right)}{Dry\;weight\;of\;the\;sample}\times 100$$

A film sample was considered disintegrated when one or more of the following conditions were observed:

- Loss of mechanical integrity such that the film could not be lifted intact with tweezers.
- Visible fragmentation, tearing, or separation into multiple pieces.
- Gel-like dissolution or softening to the extent that blotting and weighing were not feasible.
- Loss of original geometry, including deformation or dispersion preventing measurement.

Once any criterion was met, the disintegration time was recorded, and no further measurements were taken.

- **Water Solubility**

Film samples were cut into 2.0 cm × 2.0 cm squares and weighed to determine their initial mass. Each sample was immersed in a flask containing 50 mL of deionized water. The flasks were placed in a shaker and agitated at 80 rpm for 24 h at room temperature. After incubation, the solution containing the film sample was filtered using No. 1 filter paper to separate the undissolved fragments. These fragments were then dried in an oven at 105 °C overnight, and their dry weight was recorded following the method of Zinnia et al. [6]. The water solubility of the films was calculated using the following equation:

$$Water\;solubility\left(\%\right)=\frac{\left(Initial\;weight\;of\;the\;sample-weight\;of\;fragment\;after\;drying\right)}{Initial\;weight\;of\;the\;sample}\times 100$$

- **FTIR**

The Fourier transform infrared (FTIR) spectra of the biofilms were recorded using a SHIMADZU IRSpirit QATR-S FTIR spectrophotometer (SHIMADZU, Columbia, MA, USA) to characterize the functional groups and molecular interactions among the polysaccharide components. Spectral data were collected over a wavenumber range of 400–4000 cm^−1^ with 64 scans per sample to ensure an optimal signal-to-noise ratio. Measurements were performed in ATR mode using a single-reflection (single-bounce) diamond ATR crystal, which enables direct analysis of solid films without additional sample preparation. All spectra were processed and interpreted using LabSolutions IR software 2.1.

- **TGA**

The thermal analysis was performed using a thermogravimetric analyzer (Mettler Toledo, Thermal Analysis System TGA 2, Greifensee, Switzerland) to examine the thermal stability of alginate-based films. Samples of 8.3 g were subjected to heating from 25 to 600 °C in a nitrogen atmosphere at a flow rate of 50 mL min−1. All experiments were carried out at a rate of 20 °C min^−1^.

- **Microscopic Characterization:**

The microstructural characteristics of the bio-composite films were examined using a digital optical microscope (Keyence VHX-2000, Keyence Corp., Osaka, Japan). Prior to imaging, films were cut into uniform specimens (10 mm × 10 mm) from the central region to avoid edge artifacts. Surface morphology was observed under reflected light at magnifications of 80× and 150× using fixed illumination, exposure, and contrast settings for all samples to ensure comparability. Scale calibration was performed using a stage micrometer, and scale bars were automatically generated from instrument metadata. For each formulation, images were captured from at least two non-overlapping regions to ensure representative microstructural assessment. The obtained micrographs were used to qualitatively evaluate the dispersion of MAX and the presence of agglomerates, as well as the effect of CONE incorporation on matrix uniformity.

- **Anti-microbial Shelf-life Test**

The antimicrobial shelf-life assay was performed following the procedure described by Alves et al. [19]. Boneless chicken was cut aseptically into ~2.0 cm × 0.5 cm pieces (mass recorded), then wrapped with one of the following: (i) cling film (control), (ii) SA + Gly, (iii) SA + Gly + CONE, (iv) SA + Gly + MAX 1.5, or (v) SA + Gly + MAX 1.5 + CONE; an unwrapped control was included. Each piece was fully covered on both faces and edges (projected contact area ≈ 1.0 cm^2^ per face; minimum film–food contact area ≈ 2.0 cm^2^). Samples were stored for 3 and 5 days at either room temperature (22–24 °C) or 4 °C in covered sterile containers (*n* = 3 per condition). After storage, each piece was rinsed in 100 mL sterile 0.85% saline with gentle stirring for 2 min; ten-fold serial dilutions (10^−1^–10^−5^) were prepared and 0.1 mL aliquots were spread-plated (duplicate) on LB agar (Miller; Sigma-Aldrich, Waltham, MA, USA). Plates were incubated at 37 °C for 24 h and colonies counted on plates with 30–300 CFU.

### 2.4. Statistical Analysis

All the experiments were carried out in triplicates, and the results are shown as mean ± standard error. To determine statistical significance, the paired, two-tailed Student’s *t*-test was calculated using Excel.

## 3. Results and Discussion

### 3.1. Mechanical Properties

- **Tensile Strength**

**Figure 1:**
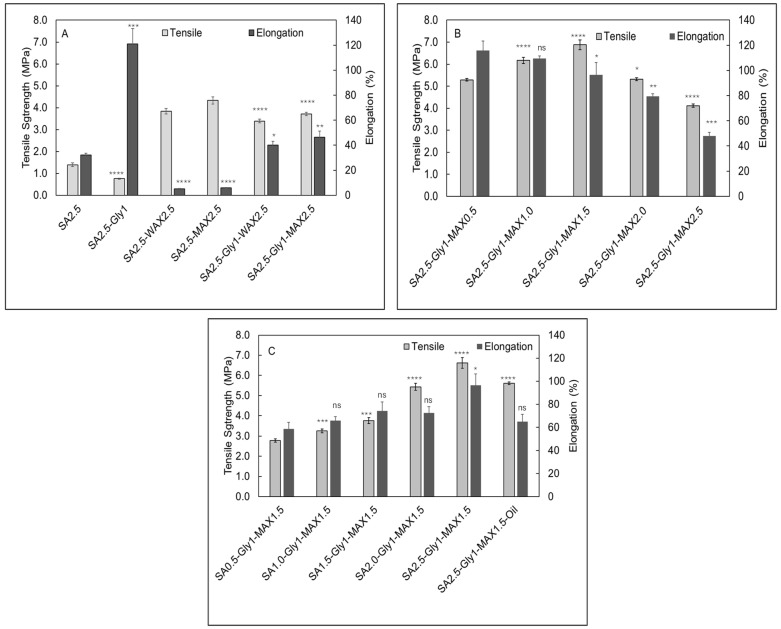
Tensile strength and elongation measurements of sodium alginate (SA)-based bio-composite films. (**A**) Comparison of film tensile strength and elongation for SA alone, SA with glycerol, and SA–glycerol films supplemented with arabinoxylan (AX). (**B**) Tensile strength and elongation of films formulated with increasing concentrations of MAX. (**C**) Tensile strength and elongation of films containing varying SA concentrations and essential oil. **Data are presented as mean ± SD (*n* = 3). *Scheme*: ns = *p* > 0.05;** * = *p* < 0.05; ** = *p* < 0.01; *** = *p* < 0.001; **** = *p* < 0.0001. **Reference groups for significance evaluation were: SA 2.5** for panel (**A**); SA 2.5-Gly 1–MAX 0.5 for panel (**B**); and SA 0.5–Gly 1–MAX 1.5 for panel (**C**).

High tensile strength and good elongation properties are requisites for manufacturing flexible and durable packaging films that can withstand external stress during food processing, transportation and storage. These properties are influenced by the method of film preparation and thickness of the resultant films [14]. The film prepared using SA alone exhibited a relatively low tensile strength of 1.39 MPa and an elongation of 32.2%. Adding glycerol into biofilm significantly improved the tensile strength to 1.73 MPa and elongation to 121.1%. To further increase the tensile strength, composite biofilms using SA and WAX or MAX were prepared. When 100 wt.% WAX and MAX were added, the tensile strength increased to 3.84 and 4.83 MPa, which were 2.8 and 3.1 times that of SA-only film, respectively (Figure 1A). However, the elongation reduced dramatically. The addition of glycerol led to a slight reduction in tensile strength and a marked increase in elongation. This occurs because glycerol acts as an effective plasticizer, weakening intermolecular hydrogen bonding and increasing polymer-chain mobility. As a result, the film becomes less rigid but more flexible. Similar trends have been reported by Syarifuddin et al. [20], who observed reduced tensile strength and enhanced extensibility in alginate-based films with increasing glycerol content. The films with MAX showed better tensile strength than the films with WAX (*p* < 0.001). This can be attributed to the different sources and extraction methods of AX and the inherent heterogenicity of AX [14,21].

To investigate the impact of AX concentration on the tensile strength, alginate films with various MAX compositions were prepared. As shown in Figure 1B, the tensile strength was 5.28 MPa when MAX concentration was 20 wt.% and increased to 6.17 MPa and 6.88 MPa when the MAX concentration was increased to 40 wt.% and 60 wt.%. However, further increases in AX concentration to 80 wt.% and 100 wt.% led to tensile strength decreasing to 5.32 MPa and 4.12 MPa. This could be due to the agglomeration of AX, making the film brittle and resulting in weakening of the film [22]. The alginate films containing 60 wt.% of MAX with various SA content were prepared to investigate the impact of SA concentration on tensile strength. These films showed an increasing trend of tensile strengths with increasing concentrations of SA (Figure 1C). The tensile strengths of the films containing 20 wt.%, 40 wt.%, 50 wt.%, 60 wt.% and 20 wt.% SA were recorded as 2.78, 3.25, 3.76, 5.43 and 6.63 MPa, respectively. These results are like findings reported by Syarifuddin et al. [20], and Zhang et al. [23], where increasing concentration of SA resulted in higher tensile strength of the films. Among all the films, SA2.5 G1y MAX1.5 had the highest tensile strength. It was selected for the incorporation of clove essential oil for the preparation of anti-bacterial biofilm. When CONE was added to this film, the tensile strength of the film reduced to 5.61 MPa (*p* < 0.001). Recently, several reports have been published for the preparation of composite SA films for food packaging applications, as summarized in Table 2. In comparison with these results, the film prepared using SA, glycerol and arabinoxylans showed promising high mechanical strength (Table 2).

- **Elongation**

The SA-only film showed relatively low elongation. However, when glycerol was added to the film, the resultant SA + Gly showed an elongation rate of ~3.75 times that of the SA film. This could be a result of the hydrogen bonding between the -COO− groups in SA matrix and the -OH group in glycerol, which reduced the crystallization in the polymeric chain, resulting in increased chain mobility [26,27]. The films with SA + WAX and SA + MAX had elongation rates of ~4.98% and ~5.86% respectively which were very low. Similarly, adding glycerol significantly increased the elongation properties of the films with AX. The SA + Gly + WAX and the SA + Gly + MAX films showed elongation rates of 40.0% and 46.4% respectively which were approximately seven times higher than that of respective films without glycerol (Figure 1A). The films prepared using SA 100 wt.% and glycerol 54 wt.% with varying MAX composition showed a decreasing trend for the elongation rate as the concentration of MAX increased (Figure 1B). The highest elongation rate of ~116% was achieved by the film with 20 wt.% MAX while the lowest elongation rate of ~48% was achieved by the film with 100 wt.% MAX. The films prepared using MAX 60 wt.% and glycerol 54 wt.% with varying SA composition showed an increasing trend for the elongation rate as the concentration of SA was increased. (Figure 1C) The lowest elongation rate of 58.5% was shown by the film with 20 wt.% SA while the highest elongation rate of 96.4% was shown by the film with 100 wt.% SA. The elongation rate was reduced when the Oil NE was added to the films, resulting in 64.8%. Films used for food packaging need to be flexible and have good elongation. When compared with biofilms reported in the literature our films (SA + Gly + MAX 2.5 and SA + Gly + MAX 2.5 + CONE) showed comparable or better elongation properties (Table 2).

### 3.2. Thickness and Visual Appearance

**Figure 2:**
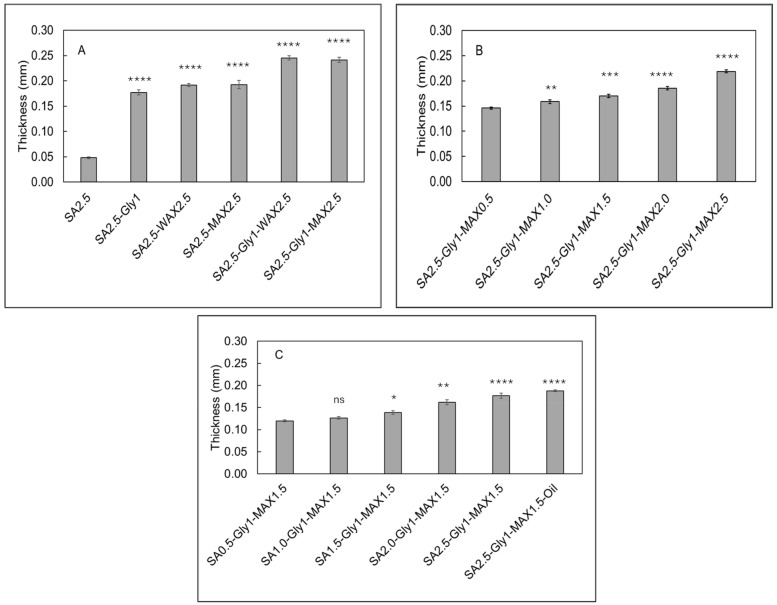
Thickness measurements of sodium alginate (SA)-based bio-composite films. (**A**) Comparison of film thickness for SA alone, SA with glycerol, and SA–glycerol films supplemented with arabinoxylan (AX). (**B**) Thickness of films formulated with increasing concentrations of MAX. (**C**) Thickness of films containing varying SA concentrations and essential oil. **Data are presented as mean ± SD (*n* = 3). *Scheme 0*. ns = *p* > 0.05;** * = *p* < 0.05; ** = *p* < 0.01; *** = *p* < 0.001; **** = *p* < 0.0001. **Reference groups for significance evaluation were: SA 2.5** for panel (**A**); SA 2.5–Gly 1–MAX 0.5 for panel (**B**); and SA 0.5–Gly 1–MAX 1.5 for panel (**C**).

The films made of SA and SA + glycerol were smooth and had uniform thickness. The average thickness of SA-only film is 0.048 ± 0.002 mm. Adding glycerol in SA increased the thickness to 0.177 mm (Figure 2A). The thickness of SA-only film is like that reported by Wang et al. [28], in which a thickness of 0.0459 ± 0.0052 mm was reported for SA-only film. However, Wang et al. [28], reported adding 1% (*w*/*v*) glycerol only led to an increase in the thickness to 0.0476 ± 0.0031 mm. While, in this study, adding glycerol significantly increases the film thickness. The thickness difference could be attributed to the different drying temperature and drying time in these two studies. The films made with WAX and MAX were smooth and showed uniform thickness. As expected, adding AX in the alginate film increased the thickness of the film. Adding glycerol further increased the thickness of the film by a factor of 26–28% (Figure 2A). The composite films made by keeping the SA and glycerol concentration constant and varying the MAX concentration were smooth and showed uniform thickness. It was observed that with increasing concentration of MAX from 20 wt.% to 100 wt.% the thickness of the films increased from 0.146 mm to 0.219 mm as seen in Figure 2B. The resulting films were white-ish in color and the opacity of the films increased with increasing concentration of MAX. Next, composite films were made by keeping the MAX and glycerol concentration constant and varying the SA concentration. The film thickness increased from 0.120 mm to 0.170 mm with an increase in SA concentration from 20 wt.% to 100 wt.% (Figure 2C). The thickness of the film with oil nano-emulsion increased slightly to 0.188 mm as compared to the film without oil nano-emulsion, which agreed with the observation reported by Mutlu et al. [29]. Oil nano-emulsion droplets tend to settle in the empty spaces in the polymeric network at lower concentration of SA. However, with an increase in concentration of constituents in the film forming solution, the oil nano-emulsion droplets do not have sufficient empty spaces to settle, they must occupy physical space and therefore contribute to the thickness of the films [30]. The thickness of the films reported in this study are in the range of 0.048 mm to 0.245 mm, which meet the ASTM D6988 standard of less than 0.25 mm [31].

### 3.3. Water and Saline Holding Capacity, Moisture Content and Water Solubility

- Water and saline holding capacity

**Figure 3:**
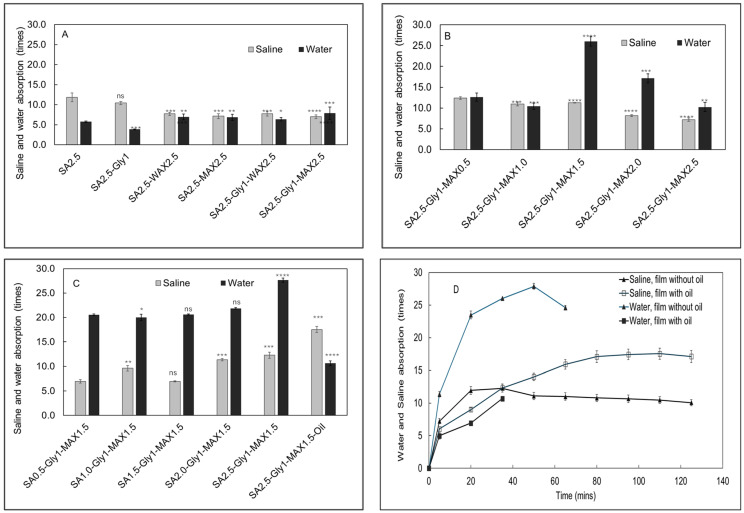
Saline and water adsorption measurements of sodium alginate (SA)-based bio-composite films. (**A**) Comparison of film saline and water adsorption for SA alone, SA with glycerol, and SA–glycerol films supplemented with arabinoxylan (AX). (**B**) Saline and water adsorption of films formulated with increasing concentrations of MAX. (**C**) Saline and water adsorption of films containing varying SA concentrations and essential oil. (**D**) Saline and water absorption time profiles for two SA films. Triangle: 2.5% SA + 1% Gly + 1.5% MAX and square: 2.5% SA + 1% Gly + 1.5% MAX + Oil. Open marker, saline absorption, filled marked, water absorption. **Data are presented as mean ± SD (*n* = 3). *Scheme 0*. ns = *p* > 0.05;** * = *p* < 0.05; ** = *p* < 0.01; *** = *p* < 0.001; **** = *p* < 0.0001. **Reference groups for significance evaluation were: SA 2.5** for panel (**A**); SA 2.5–Gly 1–MAX 0.5 for panel (**B**); and SA 0.5–Gly 1–MAX 1.5 for panel (**C**).

Alginate films have hygroscopic properties which help in absorbing and retaining moisture [32]. SA films absorb water rapidly before disintegrating and eventually dissolve in water. As shown in Figure 3A, SA films absorbed 5.7 times its own weight of water and SA + glycerol films absorbed 3.8 times its own weight of water, before disintegrating in water. For the films with various MAX concentration, the film with 60 wt.% MAX showed the highest water-holding capacity of 26 times the weight of the dry film itself (Figure 3B). Further increasing MAX content led to reduction in water-holding capacity, which may be due to the aggregation of the MAX component in the film. The films containing MAX show increased water-holding capacity with increase in SA content, ranging from 17.4 to 27.6 times the weight of the dry film (Figure 3C). The film SA + Gly + MAX 1.5 absorbed 27.6 times of water after soaking for 50 min, after which the film lost its integrity and disintegrated after 65 min (Figure 3D). When essential oil was added to the film, the water-holding capacity dropped to 10.6 and the film disintegrated after 35 min. In similar research, He et al. reported that the water-holding capacity decreased from 2.56 times to 1.94 times upon addition of essential-oil nanoparticles in the SA film [33]. The research by Jafari et al. [34] shows addition of essential oils has reduced the water-holding capacity of bio-composite films from 270% to 130% indicating that the addition of hydrophobic substances to films reduces water absorption of the films. When a saline solution was used for holding capacity test instead of water, SA film and SA + glycerol film showed significant increases in holding capacity of 11.8 and 10.4 times, respectively (Figure 3A). In a similar study by Kumar et al. [35] it has been reported that the rate of holding capacity is very high, i.e., ~8.4 times initially for SA hydrogel. For the films with various MAX concentration, the holding capacity in saline decreased from 12.4 to 7.2 times when the MAX concentration increased from 20 wt.% to 100 wt.% (Figure 3B). The films containing AX show increased holding capacity in saline with increase in SA content, although the holding capacities of saline are lower than those of water (Figure 3C). This can be attributed to the formation of the “egg-box” conformation due to the crosslinking of Ca^2+^ ions with SA, which prevents the dissolution of the films in water as reported by Costa et al. [36] Surprisingly, the film SA + Gly + MAX 1.5 + CONE showed the highest saline-holding capacity of 17.5 times. These results are like those reported by Pitterou et al. [37] where the holding capacity of the film with essential oil is 2.33 times that of the film without essential oil. In all cases, the films stay longer in the saline solution than in water. Figure 3D shows the swelling ratio profiles of two films (SA + Gly + MAX 1.5 and SA + Gly + MAX1.5 + CONE). The films kept their shape well after 2 h and remain partially integrated after 24 h.

- Moisture content

**Figure 4:**
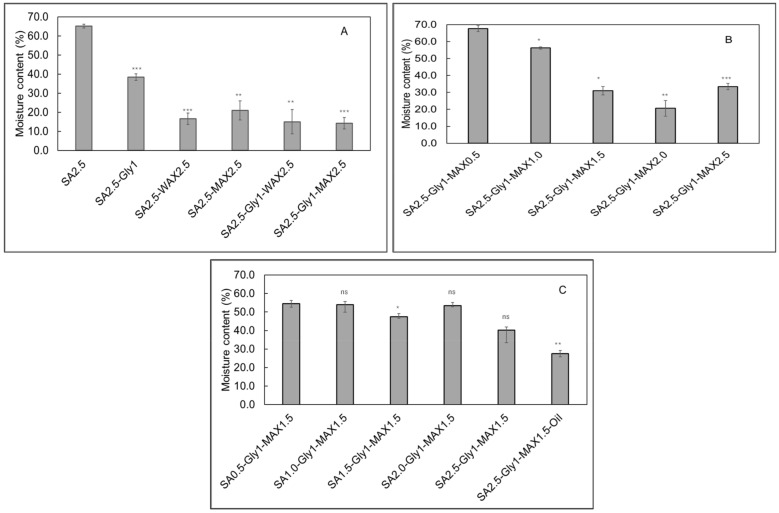
Moisture content measurements of sodium alginate (SA)-based bio-composite films. (**A**) Comparison of moisture content measurement for SA alone, SA with glycerol, and SA–glycerol films supplemented with arabinoxylan (AX). (**B**) Moisture content measurement of films formulated with increasing concentrations of MAX. (**C**) Moisture content measurement of films containing varying SA concentrations and essential oil. **Data are presented as mean ± SD (*n* = 3). *Scheme 0*. ns = *p* > 0.05;** * = *p* < 0.05; ** = *p* < 0.01; *** = *p* < 0.001. **Reference groups for significance evaluation were: SA 2.5** for panel (**A**); SA 2.5–Gly 1–MAX 0.5 for panel (**B**); and SA 0.5–Gly 1–MAX 1.5 for panel (**C**).

The moisture content of a film significantly influences the shelf life of food when utilized for packaging applications. The film having SA exhibited a moisture content of 65.2%, whereas the SA + Gly composite film demonstrated a reduced moisture content of 38.4% (Figure 4A). Films incorporating wheat arabinoxylan with SA and maize arabinoxylan with SA displayed moisture contents of 16.6% and 21.0%, respectively (Figure 4A). Upon the addition of glycerol to these composite films, the moisture content further decreased to 15.0% for film SA + Gly + WAX 2.5 and 14.2% for SA + Gly + MAX 2.5. These findings highlight the role of glycerol in modulating the hygroscopic properties of the films, thereby potentially enhancing their suitability for food packaging. For films containing 100 wt.% SA and varying concentrations of maize arabinoxylan, the moisture content ranged from approximately 20.6% to 67.7% (Figure 4B). Conversely, films formulated with 60 wt.% MAX and varying SA concentrations exhibited moisture content levels between 40.2% and 54.5% (Figure 4C). Both SA and MAX are known to possess intrinsic water retention properties, as documented in prior studies [38,39]. Consequently, the films demonstrated significantly elevated moisture content, which can be attributed to the hygroscopic nature of these biopolymers. Drying temperature and conditions show a significant influence on the moisture content and other physicochemical properties of films, as demonstrated by Wardak et al. [38]. In a related study by Harrasi et al. [39], alginate films dried at elevated temperatures exhibited reduced moisture content, highlighting the temperature-dependent nature of water evaporation during film formation. Consistent findings were reported for alginate-gelatin composite films incorporating ginger oil, where increasing the drying temperature from 25 °C to 45 °C resulted in a reduction in moisture content from 15.31 ± 0.23% to 10.12 ± 0.37% [40]. In the present study, films were dried at 40 °C in an oven overnight. When compared to the study conducted at 45 °C [41], it is plausible that the moisture content of the bio-composite films formed in this study could be further reduced by increasing the drying temperature beyond 40 °C. The film SA + Gly + MAX1.5 + CONE showed a moisture content of 27.5% which was lower than the film without oil nano-emulsion. This could be attributed to the hydrophobicity of the oil nano-emulsion, as documented by Kowalonek et al. [40]. In a study conducted by Kowalonek et al., the moisture content of the films was observed to decrease significantly from 37.96 ± 3.54% to 22.87 ± 1.39% following the incorporation of oil into the film matrix [41].

- Water Solubility

The water solubility of a film reflects its ability to dissolve in water. Due to the hydrophilic nature of SA, most SA-based films exhibit a high degree of water solubility. Upon immersion in water, SA films rapidly swell and gradually dissolve over time. Previous studies have reported that SA and SA–glycerol films become completely soluble within 24 h under standard water solubility test conditions [42]. As previously discussed in the water-holding capacity experiments, all SA-based films developed in this study lost structural integrity after 1 h of immersion in water. Water solubility was carried out to further evaluate the residual content after film dissolution. Consistent with the prior literature [43], films composed solely of SA and SA–glycerol exhibited 100% water solubility. Films incorporating WAX + SA and MAX + SA demonstrated 99.1% solubility, with the addition of glycerol having no significant impact on solubility. For films containing 100 wt.% SA and varying concentrations of MAX, water solubility slightly decreased, ranging from 97.9% to 98.7%. Similarly, films with 1.5% MAX and variable SA concentrations exhibited water solubility in the range of ~98.0% to ~98.9%. The incorporation of essential oil nano-emulsion did not alter the water solubility, which remained at 98.9%. These findings align with studies by Sucheta et al., who reported ~98% water solubility for alginate-based composite films [44]. However, contrasting results have also been documented, with lower water solubility observed for both pure-SA films and SA-based composite films. For instance, Gholizadeh et al. reported water solubility values of 84.94 ± 0.02% for pure-SA films [41], which was significantly lower than that of this study and several reported discussed above [42,43]. Additionally, Alboofetileh et al. [45] observed that composite films exhibited lower water solubility (45.40 ± 4.23%) compared to pure-SA films (81.20 ± 4.13%). These results collectively highlight the influence of composite formulation on the water solubility of SA-based films, with the inclusion of polysaccharides and other additives contributing to reduced solubility due to enhanced structural complexity and insoluble fractions.

### 3.4. FTIR Analysis

**Figure 5:**
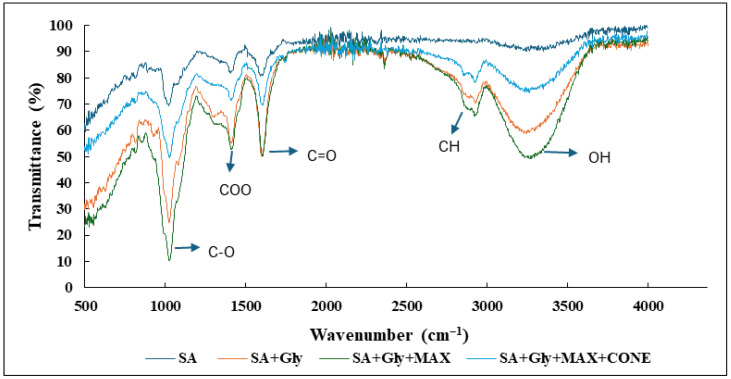
FTIR spectra of SA, SA + Gly, SA + Gly + Corn AX, and SA + Gly + Corn AX + CONE bio-composite films. Characteristic absorption bands corresponding to C–O stretching, COO– asymmetric stretching, C=O vibrations, CH stretching, and O–H stretching are indicated. Shifts in peak positions and variations in peak intensity reflect the interactions between SA, glycerol, arabinoxylan, and essential oil components within the film matrix.

The FTIR spectra of the composite films reveal clear evidence of molecular interactions among sodium alginate (SA), glycerol (Gly), MAX, and CONE, as demonstrated through systematic peak shifts and changes in band intensities (Figure 5). All films showed a broad O–H stretching band between 3000 and 3400 cm^−1^, but the progressive red-shift from ~3350 cm^−1^ in pure SA to ~3230 cm^−1^ in the SA + Gly + MAX + CONE film indicates increasingly stronger hydrogen bonding within the polymer matrix. This trend supports the enhanced intermolecular interactions introduced by plasticization (Gly) and incorporation of MAX and CONE. Similar peak positions for hydroxyl groups have been reported for SA-based films by Asmadi et al. and Chen et al., confirming the expected behavior of the polysaccharide backbone [46,47]. Shifts in the C=O band (1700–1600 cm^−1^) from ~1620 cm^−1^ in SA to ~1600 cm^−1^ in the quaternary system further suggest involvement of carbonyl groups in coordination or hydrogen-bonding interactions, consistent with observations by Mohamadi et al. for SA films containing essential oils. Likewise, the COO^−^ asymmetric stretch (~1400 cm^−1^) and the C–O stretching region (~1100 cm^−1^) exhibited noticeable downshifts and reduced intensities as additional components were incorporated, indicating modifications in the alginate carboxylate environment and formation of new linkages. These spectral changes collectively confirm that compositional additions alter the molecular arrangement of the matrices, promoting stronger associative interactions and resulting in more integrated and structurally modified composite films. This aligns with earlier FTIR reports for modified SA systems, including those by Cheng et al., and reinforces that MAX and CONE actively participate in chemical and hydrogen-bonding interactions within the film network [48].

### 3.5. TGA

**Figure 6:**
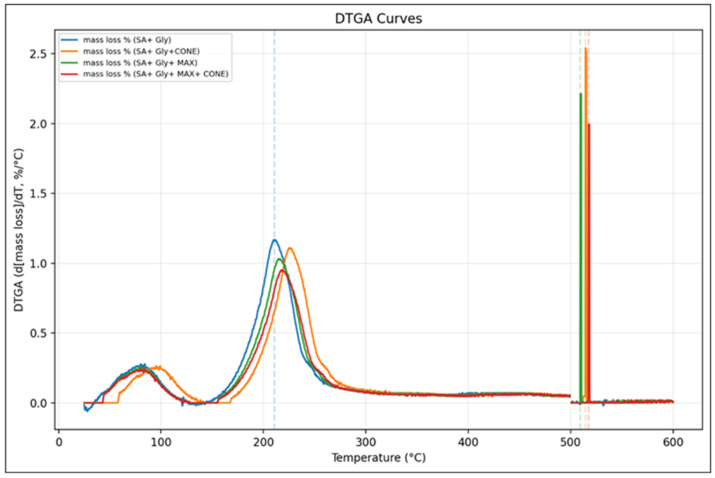
Derivative thermogravimetric (DTGA) curves of SA − Gly, SA − Gly + CONE, SA − Gly + MAX, and SA − Gly + MAX + CONE biopolymer films. The incorporation of MAX and CONE results in significant high-temperature shifts in the DTGA peaks (>500 °C), indicating enhanced thermal stability and char-forming behavior compared to the neat SA − Gly matrix.

**Figure 7:**
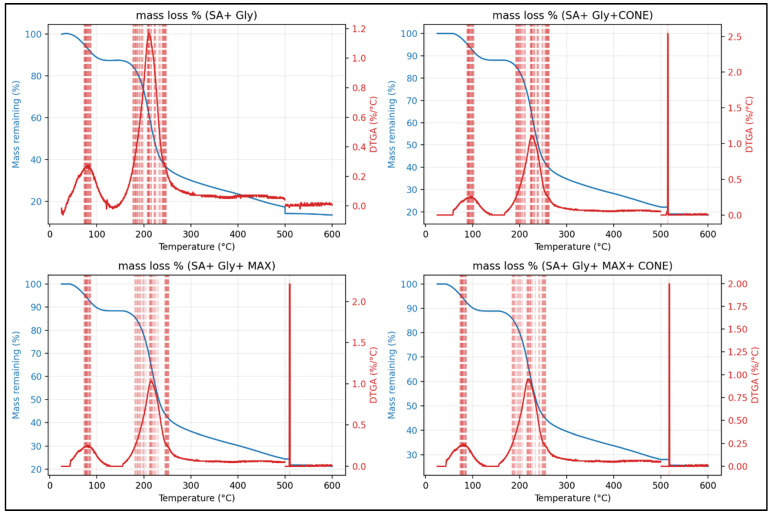
TGA and DTGA overlay plots for each material formulation. The left axis represents mass remaining (%), while the right axis depicts the derivative of mass loss (DTGA). The multi-panel layout highlights the differences in degradation pathways, with MAX + CONE showing the broadest and highest-temperature decomposition peaks.

**Figure 8:**
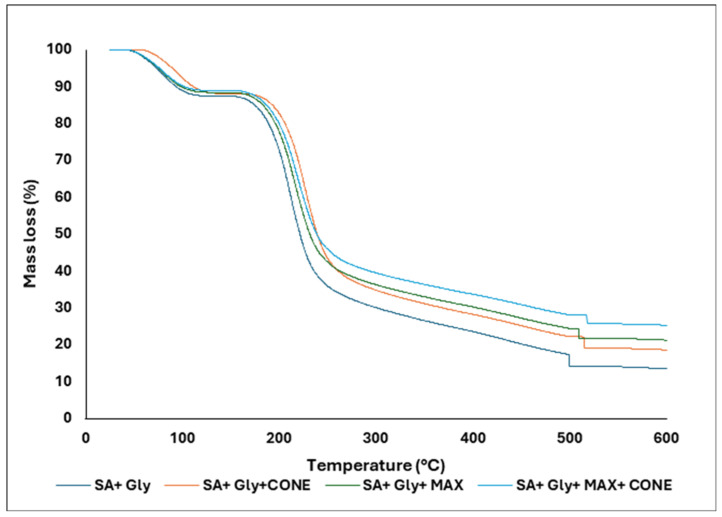
Thermogravimetric analysis (TGA) SA + Gly, SA + Gly + CONE, SA + Gly + MAX, and SA + Gly + MAX + CONE films.

The thermal behavior of SA–Gly films and their composites is summarized in Table 3, which shows that incorporating CONE or MAX significantly enhances the stability of the alginate matrix. The neat SA–Gly film decomposed early (Td5% = 72.6 °C) with a single DTGA maximum at 210.7 °C and a low char residue of 13.5%, indicating limited thermal robustness. In contrast, both CONE- and MAX-containing films exhibited delayed degradation, with Tmax values shifting into the high temperature region (509–515 °C) and char yields increasing to 18.6–21.2%, reflecting stronger condensed phase reinforcement and enhanced carbonization. The MAX + CONE composite showed the greatest improvement (Tmax = 517.5 °C; residue = 25.2%), confirming a synergistic stabilizing effect on the alginate network. These trends are clearly supported by the DTGA curves (Figure 6) and multi panel TGA–DTGA plots (Figure 7), which show suppressed low temperature volatilization, broader high temperature transitions, and higher residual mass in all additives containing films, demonstrating a shift toward a slower, char-rich degradation pathway that enhances high temperature durability. Complementarily, TGA (Figure 8) further confirms that MAX and CONE raise the onset of major degradation (from 150 °C in SA + Gly to 165–170 °C in modified films) and reduce overall mass loss, aligning with reports in the literature showing that natural bioactive additives can elevate thermal resistance in biopolymer films. Bhatia et al. [49] reported that SA and casein-based films enhanced with sage essential oil exhibited thermal stability up to 120 °C. Similarly, Harrasi et al. [39] found that ginger oil-infused gelatin-sodium alginate edible films maintained thermal stability up to 150 °C. These studies support the assertion that the thermal stability temperature of 170 °C for the SA + Gly + MAX + CONE film in the current study is superior to that of the others. Collectively, these improvements highlight the suitability of the modified films for food-packaging applications requiring enhanced thermal stability, durability, and consumer-friendly, clean-label performance. The temperatures above 600 °C were not used to avoid crucible and furnace artifacts common in biopolymer systems.

### 3.6. Microscopic Characterization

**Figure 9:**
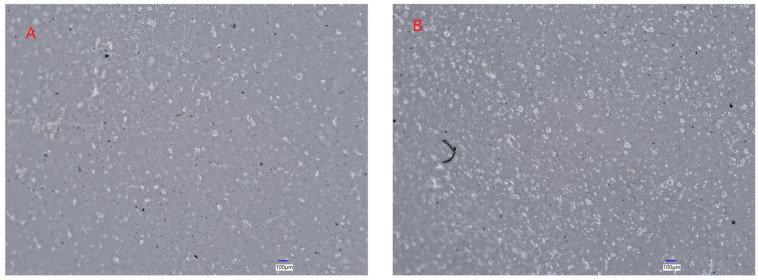
Optical micrographs of sodium alginate-based composite films captured using a Keyence Digital Microscope (VHX-2000, Keyence Co., Osaka, Japan). (**A**) SA + Gly + MAX film displaying a uniform and continuous surface morphology with finely dispersed domains, indicating effective dispersion of MAX within the alginate matrix. (**B**) SA + Gly + MAX + CONE film showing larger bright particulates and clustered domains, evidencing MAX agglomeration induced by essential-oil incorporation. Such aggregation disrupts matrix uniformity and corresponds to the reduced mechanical strength observed for this formulation. Scale bar: 100 µm.

Microscopic examination revealed clear morphological differences between formulations. The SA + Gly + MAX film showed a uniform and continuous structure with finely dispersed domains, indicating good compatibility and effective MAX dispersion within the alginate matrix (Figure 9A). In contrast, the SA + Gly + MAX + CONE film displayed larger bright particulates and clustered domains, demonstrating MAX agglomeration induced by essential-oil incorporation (Figure 9B). Such aggregation disrupts matrix uniformity and creates stress-concentration points, consistent with the observed reduction in tensile strength [20,23]. To understand these findings in comparison to commercial packaging, recent work comparing biodegradable films with commercial plastics reports that materials such as low-density polyethylene (LDPE) exhibit highly uniform, aggregation-free microstructures that contribute to their superior mechanical stability [50]. Although our films show lower mechanical values than synthetic plastics, the optimized SA + Gly + MAX formulation demonstrates competitive strength within the biodegradable category. The observed agglomeration highlights a key barrier to matching commercial film performance and underscores the importance of controlling MAX dispersion to further enhance the mechanical reliability of alginate-based packaging films.

### 3.7. Anti-Microbial Shelf-Life Assessment

**Figure 10:**
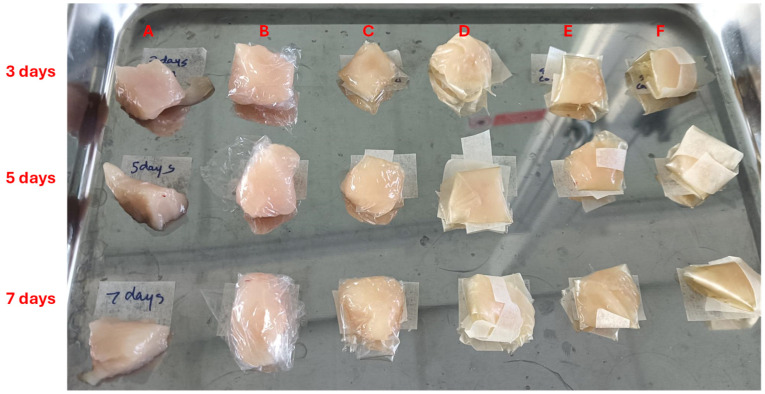
Visual assessment of chicken pieces packaged using different film formulations over storage at room temperature (3, 5, and 7 days). (**A**) Unwrapped control; (**B**) commercial cling film; (**C**) 2.5% sodium alginate (SA) + 1% glycerol (Gly); (**D**) 2.5% SA + 1% Gly + 1.5% MAX; (**E**) 2.5% SA + 1% Gly + 0.5% CONE; (**F**) 2.5% SA + 1% Gly + 1.5% MAX + 0.5% CONE.

Selected films were tested for anti-microbial shelf-life assessment (Figure 10). Bacterial colony forming unit (CFU) count after 72 and 120 h depicted that bio-composite films having clove essential oil inhibit the bacterial growth completely both at room and refrigerated (4 °C) temperature because clove oil possesses strong antimicrobial properties (Table 4) [51]. Contrary to present study where 100% inhibition of microbial growth was achieved, Upadhye et al. [52] reported 96.74 ± 5.13% and 96.13 ± 3.41% inhibition against *S. aureus* and *E. coli*, respectively, using sodium alginate + gelatin + clove oil films. Cling film wrapping restricted the microbial growth to 3.9 × 10^6^ CFU and 3.7 × 10^6^ CFU at room temperature and 4 °C after 3 days which was reached to 6.8 × 10^7^ CFU and 4.5 × 10^7^ CFU after 5 days. Microbial restriction in meat samples wrapped in cling film may be attributed to introduction of physical barrier between atmospheric air and meat which reduces the microbial contact with the meat [53]. Whereas SA + Gly and SA + Gly + MAX films proved less efficient in restricting microbial growth in comparison to cling film. The presence of alginate and MAX in the films acts as an excellent site for microbial adhesion and a carbon source, which might have helped the microbial growth, as evident from the results. The films with such polysaccharides should be supplemented with anti-adhesive substances (chitosan) or anti-microbial components (silver nanoparticles or essential oils) for their effective application in food packaging [54,55]. The same was monitored in present study, with no growth detected in the meat suspension samples which were wrapped in SA + Gly + CONE and SA + Gly + MAX + CONE films. Therefore, bio-composite films with essential oil can be considered as effective in enhancing the shelf life of meat by reducing microbial contamination. The high initial CFU/g values are consistent with raw, unpackaged supermarket chicken, which commonly contains 10^5^–10^7^ CFU/g due to natural surface contamination during processing, storage, and handling [56]. The unwrapped control in our study therefore reflects real-world microbial loads rather than artificially clean laboratory meat.

## 4. Conclusions

In conclusion, this study shows that sodium alginate-based bio-composite films reinforced with maize arabinoxylans possess improved mechanical performance and enhanced water- and saline-holding capacities. The incorporation of 60 wt.% MAX notably increased tensile strength and elongation, while the addition of clove essential oil introduced measurable antimicrobial activity. Although these results indicate that MAX- and CONE-modified films offer functional advantages over neat SA − Gly films, their applicability to food packaging should be considered preliminary. Further evaluation, particularly of barrier properties, shelf-life performance, and real-food interactions, is required to fully establish their suitability for commercial use. Additionally, OTR evaluation, detailed SEM morphology, and EO release kinetics will be carried out in future work to provide a more comprehensive mechanistic understanding of the film performance. Overall, the findings demonstrate the potential of these bio-composites as promising candidates for sustainable packaging solutions, while highlighting the need for additional functional and safety assessment.

## Figures and Tables

**Table 1:** Composition of biofilm formulations expressed as weight percentage (wt.%) relative to sodium alginate (SA). An amount of 2.5 g SA per 100 mL film-forming solution; glycerol density = 1.26 g/mL; CONE density ≈ 1.04 g/mL.

Sample Code	SA (g)	Glycerol (g)	MAX/WAX (g)	CONE (g)	Additive wt.% Relative to SA
SA	2.50	–	–	–	–
SA − Gly	2.50	1.26	–	–	Gly = 50.4 wt.%
SA − WAX	2.50	–	2.50	–	WAX = 100 wt.%
SA − MAX	2.50	–	2.50	–	MAX = 100 wt.%
SA − Gly -WAX	2.50	1.26	2.50	–	WAX = 100 wt.%; Gly = 50.4 wt.%
SA + Gly + MAX	2.50	1.26	2.50	–	MAX = 100 wt.%; Gly = 50.4 wt.%
SA − Gly − MAX	2.50	1.26	0.50	–	MAX = 20 wt.%; Gly = 50.4 wt.%
SA − G1y − MAX1.0	2.50	1.26	1.00	–	MAX = 40 wt.%; Gly = 50.4 wt.%
SA − G1y − MAX1.5	2.50	1.26	1.50	–	MAX = 60 wt.%; Gly = 50.4 wt.%
SA − G1y − MAX2.0	2.50	1.26	2.00	–	MAX = 80 wt.%; Gly = 50.4 wt.%
SA − G1y − MAX2.5	2.50	1.26	2.50	–	MAX = 100 wt.%; Gly = 50.4 wt.%
SA − Gly − MAX	0.50	1.26	1.50	–	MAX = 300 wt.%; Gly = 252 wt.%
SA1 − G1y − MAX1.5	1.00	1.26	1.50	–	MAX = 150 wt.%; Gly = 126 wt.%
SA1.5 − G1y − MAX1.5	1.50	1.26	1.50	–	MAX = 100 wt.%; Gly = 84 wt.%
SA2 − G1y − MAX1.5	2.00	1.26	1.50	–	MAX = 75 wt.%; Gly = 63 wt.%
SA2.5 − G1y − MAX1.5	2.50	1.26	1.50	–	MAX = 60 wt.%; Gly = 50.4 wt.%
SA − Gly − CONE	2.50	1.26	–	0.50	CONE = 20 wt.%; Gly = 50.4 wt.%
SA − Gly − MAX2.5 − CONE	2.50	1.26	1.50	0.50	MAX = 60 wt.%; CONE = 20 wt.%; Gly = 50.4 wt.%

**SA**: sodium alginate; **Gly**: glycerol; **WAX**: wheat arabinoxylan; **MAX**: maize arabinoxylan; **CONE**: clove oil nano-emulsion.

**Table 2:** Mechanical properties of SA bio-composite films for food packing.

Materials	Tensile Strength (MPa)	Elongation (%)	Application	Ref.
SA + Pectin + Cassia Essential Oil	5.84 ± 0.23	122.87 ± 3.89	Food packaging	[9]
SA + Pectin + Cinnamic acid	0.124 ± 0.005	13.9 ± 0.1	Food Packaging	[24]
SA + Gelatin + cellulose SA + Gelatin + cellulose+ *Boswellia sacraoleo* gum resin	6.67 ± 0.59 1.38 ± 0.13	83.50 ± 3.94 57.42 ± 3.74	Food Packaging	[10]
SA + Japanese Rice Vinegar + Peppermint Oil	0.00166	15 ± 2	Food Packaging	[25]
SA + carboxymethyl cellulose-sodium salt (NaCMC)	2.51	61	Food Packaging	[26]
SA + Gly + MAX 2.5 + CONE	5.61 ± 0.26	64.8 ± 6.5	Food packaging	This study
SA + Gly + MAX 2.5	6.63 ± 0.06	96.4 ± 9.9	Food packaging	This study

**Table 3:** Thermogravimetric parameters of SA–Gly films and modified composites.

Sample	Td5% (°C)	Tmax (°C)	Residual Mass (%)
SA + Gly	72.6	210.7	13.5
SA + Gly + CONE	89.5	514.5	18.6
SA + Gly + MAX1.5	75.2	509.5	21.2
SA + Gly + MAX1.5 + CONE	76.2	517.5	25.2

**Table 4:** Total viable bacterial count of meat stored under different conditions.

Packaging	Day 0 CFU/g	Day 3 CFU/g		Day 5 CFU/g	
	Control	Room Temperature	Refrigerator (4 °C)	Room Temperature	Refrigerator (4 °C)
No wrapping	3.1 ± 0.02 × 10^6^	4.1 ± 0.04 × 10^6^	3.7 ± 0.03 ×10^6^	4 ± 0.03 × 10^9^	2 ± 0.07 × 10^9^
Cling Film	-	3.9 ± 0.01 × 10^6^	3.7 ± 0.01 × 10^6^	6.8 ± 0.03 × 10^7^	4.5 ± 0.04 × 10^7^
SA + Gly	-	2.1 ± 0.07 × 10^6^	1 ± 0.06 × 10^6^	1.5 ± 0.01 × 10^8^	8.7 ± 0.01 × 10^7^
SA+ Gly + MAX1.5	-	3.5 ± 0.02 × 10^6^	1.5 ± 0.01 × 10^6^	1.1 ± 0.05 × 10^8^	8 ± 0.04 × 10^7^
SA + Gly + CONE	-	No growth	No growth	No growth	No growth
SA + Gly + MAX1.5 + CONE	-	No growth	No growth	No growth	No growth

## Data Availability

The original contributions presented in this study are included in the article/Supplementary Material. Further inquiries can be directed at the corresponding author.

## Supplementary Materials

The following supporting information can be downloaded at: https://www.mdpi.com/article/10.3390/foods15061035/s1, Figure S1: Visual appearance of solvent-cast sodium-alginate bio-composite films.

## Abbreviations

The following abbreviations are used in this manuscript:

SA	Sodium Alginate
Gly	Glycerol
WAX	Wheat Arabinoxylan
MAX	Maize Arabinoxylan
CONE	Clove oil nano-emulsion
