# Supplementary material for: Alginate–Arabinoxylan Composite Films with Enhanced Mechanical Strength and Functional Properties for Potential Food Packaging Use
Source: Foods. 2026 Mar 16;15(6):1035. doi: 10.3390/foods15061035 (PMC13025781; doi:10.3390/foods15061035)
Supplement: Supplementary file 1 [file foods-15-01035-s001.zip › foods-4181615-supplementary.pdf]

Shelf Life

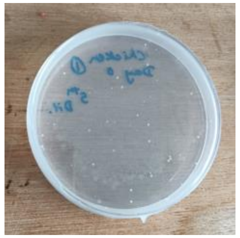 Day 0

Day 3

Outside

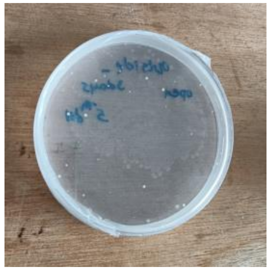 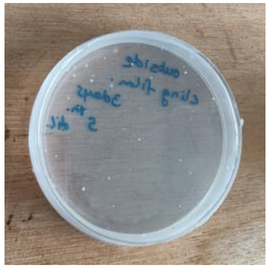 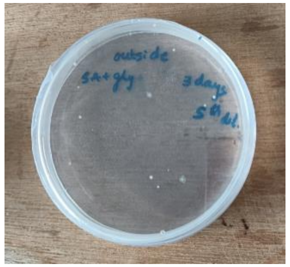 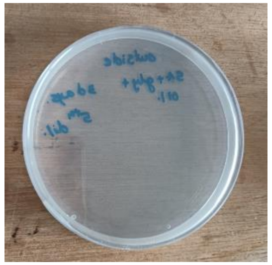 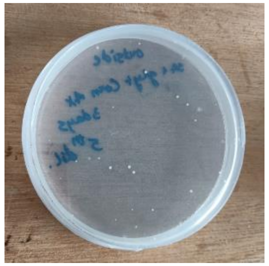 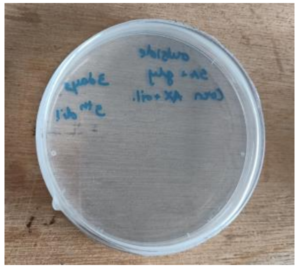

Left to Right: Open, Cling Film, SA+ Gly, SA+ Gly+ Oil, SA+ Gly+ Corn Ax, SA+ Gly+ Corn Ax+ Oil

Fridge

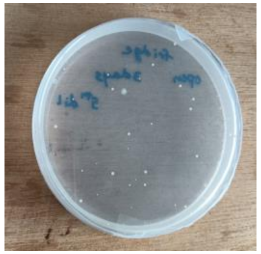 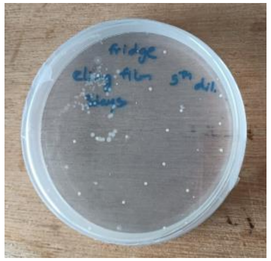 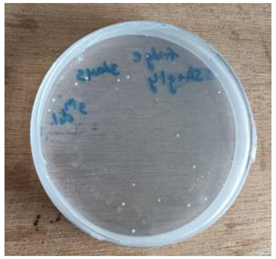 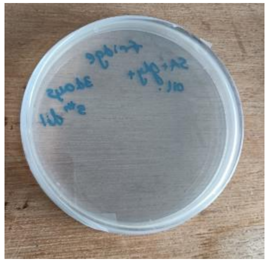 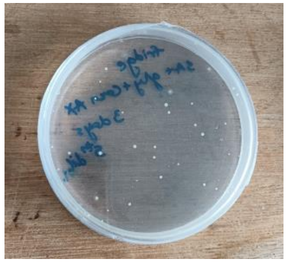 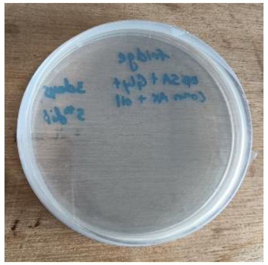

Left to Right: Open, Cling Film, SA+ Gly, SA+ Gly+ Oil, SA+ Gly+ Corn Ax, SA+ Gly+ Corn Ax+ Oil

5 Days

Outside

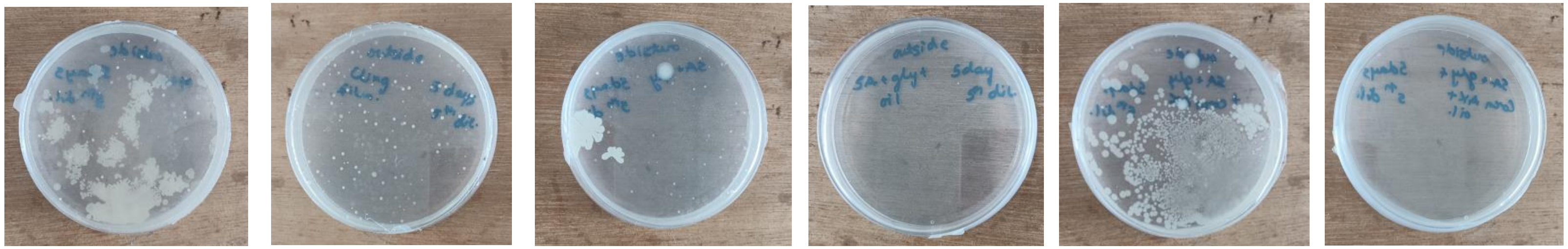

Left to Right: Open, Cling Film, SA+ Gly, SA+ Gly+ Oil, SA+ Gly+ Corn Ax, SA+ Gly+ Corn Ax+ Oil

Fridge

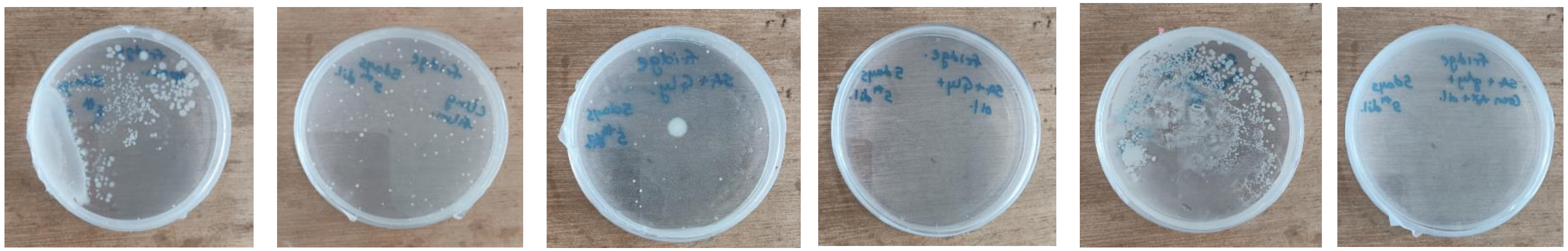

Left to Right: Open, Cling Film, SA+ Gly, SA+ Gly+ Oil, SA+ Gly+ Corn Ax, SA+ Gly+ Corn Ax+ Oil

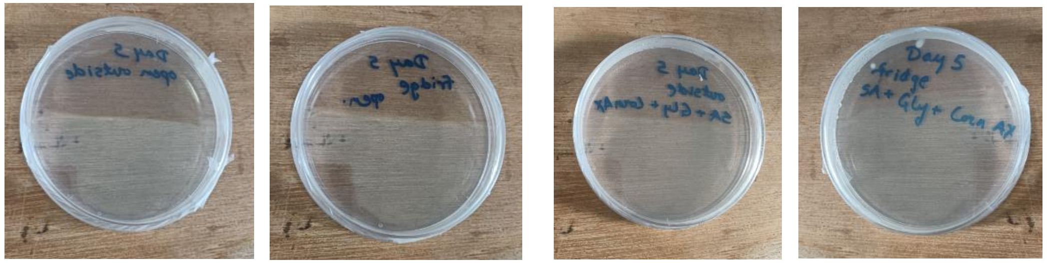

Left to Right: Day 5 (10^{-9} dilution) Open: Outside, Fridge, (10^{-7} dilution) SA+ Gly+ Corn AX: Outside, Fridge

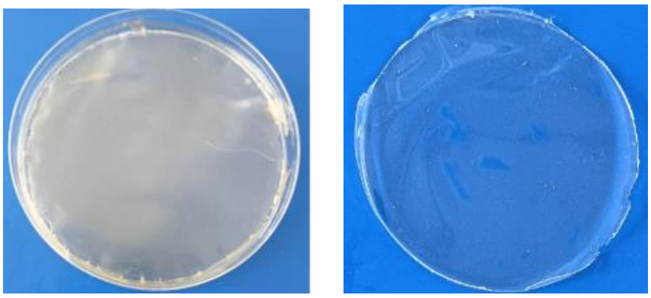

Figure S1: Visual appearance of solvent-cast sodium-alginate bio-composite films
